# Uncomplicated full-term birth after laparoscopical salpingotomy as organ-preserving therapy of naturally conceived heterotopic pregnancy: A case report

**DOI:** 10.1177/2050313X251325121

**Published:** 2025-03-12

**Authors:** Roland Csorba, Antonella Iannaccone, Panagiotis Tsikouras, Saed Almasarweh

**Affiliations:** 1 Department of Obstetrics and Gynecology, University Clinic, University of Duisburg-Essen, Essen, Germany; 2 Department of Obstetrics and Gynecology, Democritus University of Thrace, Komotini, Greece

**Keywords:** Heterotopic pregnancy, naturally conceived pregnancy, laparoscopy, full-term birth

## Abstract

Heterotopic pregnancy, the simultaneous occurrence of intrauterine and extrauterine gestations, is a rare phenomenon, especially without a history of assisted reproduction. This case report describes a 33-year-old woman presenting with severe abdominal pain at 7 weeks of pregnancy. Transvaginal ultrasound revealed an intrauterine singleton pregnancy and an adnexal mass, highly suspicious of an ectopic pregnancy, along with significant intraperitoneal fluid. Emergency laparoscopy confirmed intra-abdominal hemorrhage and a near rupture of the left fallopian tube. A laparoscopic salpingotomy successfully resolved the ectopic pregnancy while preserving the intrauterine pregnancy. Postoperative course was uneventful. The patient later delivered a healthy infant at term. This case highlights the importance of considering heterotopic pregnancy in patients with confirmed intrauterine pregnancy, even in the absence of assisted reproductive technologies and other risk factors.

## Introduction

Heterotopic pregnancy (HP) refers to the coexistence of an intrauterine pregnancy (IUP) and an ectopic pregnancy (EP), with incidences ranging from 1 in 30,000 spontaneous pregnancies to 1%–2% in assisted reproduction cases.^1–3^ HP implies that two or more implantation sites co-occur. The word heterotopic arises from the Greek language, in which “hetero” means other and “topos” means place.^3^ Despite advances in medical technology and diagnostic methods, HP presents significant challenges in both diagnosis and management. Most ectopic pregnancies occur in the fallopian tube, although implantation can occur in the cervix, cesarean scar, ovary, or abdominal cavity.^4–6^ The condition is associated with increased maternal morbidity and mortality, making early diagnosis and appropriate management crucial to optimizing maternal and obstetrical outcomes.

In addition, HP is challenging to diagnose as the IUP can obscure the ectopic component. Here, we describe a case of a naturally conceived HP in a 33-year-old patient with the successful treatment of an EP with the preservation of fallopian tubes and the intrauterine (IU) embryo, which resulted in a spontaneous full-term vaginal birth.

## Case presentation

A 33-year-old female (gravida 3 para 1) presented to our tertiary hospital complaining of weakness, presyncope, and acute lower abdominal and epigastric pain. The pain started 1 day prior to presentation without nausea, vomiting, other gastrointestinal symptoms, or vaginal bleeding. Her past medical history was unremarkable. Her past obstetric history revealed an early abortion in the first trimester and a full-term normal vaginal delivery.

Vital signs revealed tachycardia (110 beats/min), normal blood pressure, and normal body temperature. Physical examination revealed tenderness in the lower abdomen with palpation.

**Figure 1.**
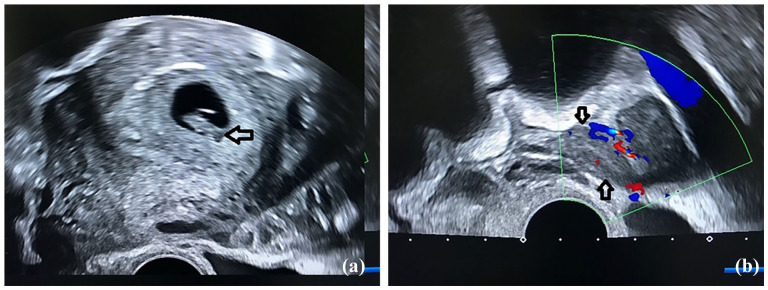
(a) Transvaginal ultrasonography (TVUS) revealed a single intrauterine (IU) gestation with a crown–rump length (CRL) of 12 mm (Black arrow) with a positive fetal heart rate and (b) A suspected ectopic gestation (Black arrows) in the left fallopian tube with positive Doppler ultrasound peripherally.

Transvaginal ultrasonography (TVUS) revealed a single IU gestation with a crown–rump length (CRL) of 12 mm, a positive fetal heart rate, and a suspected ectopic gestation in the left fallopian tube (Figure 1(a) and (b)). The contralateral ovary and fallopian tube were unremarkable. TVUS also demonstrated a moderate amount of free intraperitoneal fluid in the pouch of Douglas. Booking laboratory tests showed a reduced hematocrit of 0.26% and a serum hemoglobin concentration of 9.2 g/dL, along with a normal blood platelet level. The serum β-human chorionic gonadotropin level was 88,635 mIU/mL.

**Figure 2.**
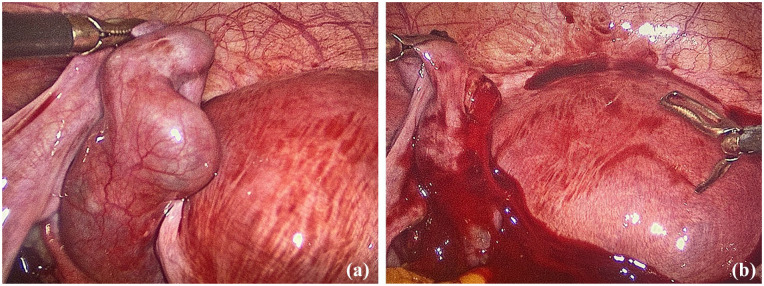
(a) Extrauterine gravidity in the left fallopian tube, just before rupture. Moderate amount of free intraperitoneal fluid in the pouch of Douglas and (b) Salpingotomy of the left fallopian tube. The ectopic pregnancy was successfully removed and both of the fallopian tubes were preserved.

After thorough counseling and receiving the patient’s consent, an emergency laparoscopy was performed under general anesthesia. Intraoperatively an estimate of 400 mL blood was evacuated from the peritoneal cavity from a partially ruptured left fallopian tube, thus confirming the diagnosis of HP. Attending to the patient’s request for organ-preserving surgery, a left laparoscopic salpingotomy was performed (Figure 2(a) and (b)). The EP was successfully removed, and both of the fallopian tubes were preserved. A histological examination confirmed chorionic villi suggestive of an approximately eighth-week EP. There were no complications during the postoperative course.

**Figure 3.**
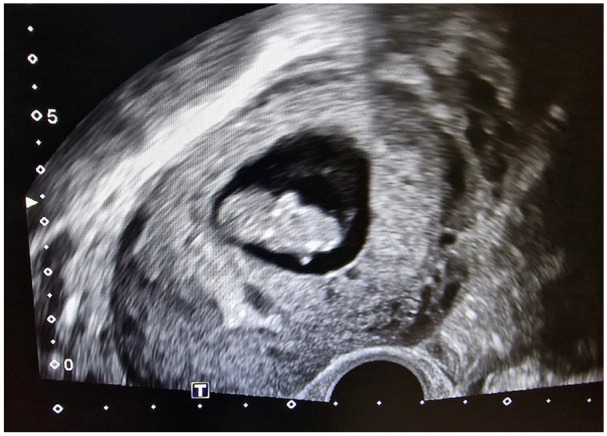
Postoperative TVUS: IU embryo with a CRL of 38 mm, 9 + 2 weeks of pregnancy.

Postoperatively the patient received progesterone support (200 mg/day intravaginally) and the supportive progesterone therapy was continued until the 12th gestational week. The patient recovered well and was discharged from the hospital on the third postoperative day. She had regular antenatal care appointments, and her pregnancy was uneventful (Figure 3). The development of the fetus was normal, and at 39 weeks and 5 days of gestation, the patient gave normal vaginal birth to a healthy boy who was 52 cm tall and weighed 3240 g (Apgar 9/10/10, umbilical cord pH = 7.26). Postnatal recovery was without any complications, and the patient was discharged on the third postpartum day.

## Discussion

HP is defined as an uncommon condition when IU and extrauterine gestations coexist, and it was first described in 1708 by Duverney, who recognized and described the disease during an autopsy.^7^ After the Second World War, more papers were published, mostly by American authors.^8,9^

Despite its rarity, its incidence has risen with the increased use of assisted reproductive technologies and ovarian stimulation for infertility treatment.^10^ Pelvic inflammatory disease, previous pelvic surgery, and tubal damage are classified as the most important risk factors for HP.^11,12^ However, the presented patient had a spontaneous pregnancy without the above-mentioned risk factors, thereby making our case even more unique.

Location of the EP is mostly in the fallopian tube (95%), in the cervix uteri, in a scar of a previous cesarean section, in the ovary, and in the abdominal cavity. In our case, the disease EP affected the left fallopian tube of our patient.

Majority of all HP cases are diagnosed between the 5th and 8th weeks of pregnancy and only 10% after the second trimester.^12^ The symptomatology of HP can vary widely, as a quarter of the cases have no symptoms. The most common symptoms include abdominal or pelvic pain and vaginal bleeding. Hypovolemic shock and acute abdomen may indicate an abdominal disaster. Occasionally, the IU pregnancy is diagnosed after performing an emergency operation on a ruptured EP, which makes the differential diagnosis difficult. During pregnancy care, especially during the first ultrasound examination of the patient, it is important to remember that the presence of an IU pregnancy does not exclude the presence of a simultaneous EP and an IU pregnancy may exist in addition to an EP.^13,14^ It is, therefore, necessary to try to depict the uterus and both adnexa to recognize a possible HP in time. For this purpose, it is advisable to supplement the transvaginal ultrasound most often used in early pregnancy with a transabdominal ultrasound, although in our case, in addition to the early pregnant uterus, the adnexa were also correctly depicted during the transvaginal ultrasound examination. This is also supported by the fact that TVUS has been found to be better in early diagnosis compared to transabdominal US. It detects almost 70% of cases between the fifth and eighth weeks of gestation.^14^

The management of HP remains controversial. Treatment experiences are restricted and there is minor consensus regarding optimal management.^15^ The aim is to terminate the EP while minimizing harm to the mother and IU fetus. Management of HP depends on the clinical situation, the patient’s stability, the size and site of an EP, previous pregnancies, family planning, and the viability of both pregnancies. Treatment options include surgical intervention, ultrasound-guided embryo aspiration with or without local embryo-killing drugs, and expectant management.^16,17^ Non-surgical treatments involve transabdominal ultrasound-guided transvaginal aspiration of the EP with or without direct injection of agents such as potassium chloride (KCl) or hyperosmolar glucose.^16^ Methotrexate, generally used in a single EP, is contraindicated due to the toxicity of the IU fetus.^18^ Expectant management with frequent follow-up ultrasound examinations and close observations should only be considered in hemodynamically stable symptom-free patients.^19^

For patients, like ours, with signs of hypovolemic shock or in the case of the threatening rupture of EP, an emergency operation is strongly indicated. The advantage of surgery is the ability to completely remove the EP, the disadvantage is the higher abortion rate of an IU embryo. In our case, an urgent left laparoscopic salpingotomy was chosen due to the free intraperitoneal blood in the pouch of Douglas and the suspicion of the rupture of the EP. Both fallopian tubes were preserved according to the clear request of the patient. Contrary to our decision, different recommendations are also found in the literature. A Canadian guideline underlined that there is no evidence to recommend conservative, tube-sparing salpingotomy over salpingectomy when the contralateral fallopian tube is normal.^20^ Our patient opted for organ-preserving surgery, which was successfully performed without affecting the patient’s outcome.

## Conclusion

HP is a rare but potentially life-threatening condition requiring early detection and intervention. Despite advancements in diagnostic imaging and treatment, HP remains challenging due to its nonspecific presentation and rarity. This case highlights the importance of considering HP in pregnant patients with acute abdominal pain, even in those with confirmed IU pregnancies. Prompt diagnosis and appropriate management are critical for successful maternal and fetal outcomes.
